# Influence of Perceived Environmental Quality on the Perceived Restorativeness of Public Spaces

**DOI:** 10.3389/fpsyg.2021.644763

**Published:** 2021-04-16

**Authors:** María Luisa Ríos-Rodríguez, Christian Rosales, Maryurena Lorenzo, Gabriel Muinos, Bernardo Hernández

**Affiliations:** ^1^Department of Social Psychology, Social Work, Social Anthropology and E.A.O., Universidad de Málaga, Málaga, Spain; ^2^Department of Cognitive, Social and Organizational Psychology, Universidad de La Laguna, San Cristóbal de La Laguna, Spain; ^3^Department of Health Sciences, Universidad Europea Miguel de Cervantes, Valladolid, Spain; ^4^Department of Psychology, University of Groningen, Groningen, Netherlands

**Keywords:** restorativeness, public space, environmental quality, parks, town squares

## Abstract

Parks and town squares can play an important role by offering spaces for cognitive restorativeness in urban contexts. Therefore, it is important that these spaces be designed in a way that encourages restorativeness. Indeed, their perceived quality should motivate users to stay and take advantage of them. Yet, it is not clear whether perceptions as to the quality of these spaces is relevant in promoting restorativeness. Thus, the aim of this study is to analyze whether elements of environmental quality perceived by users of public spaces favor restorativeness both in parks and squares. Environmental and social aspects are taken into consideration, since restorative experiences involve cognitive and physiological recovery, as well as a component of interaction with the environment. In this research, 519 users of 32 urban public spaces—town squares and parks—on the island of Tenerife (Spain) participated. Participants evaluated these spaces using four dimensions that focused on spaces’ perceived environmental quality: design of spaces, care of spaces, social interaction, and presence of sensorial elements. Additionally, we evaluated the perceived restorativeness of each space. The results showed that the design of spaces, care of the spaces, social interaction, and presence of sensorial elements explain the variance in perceived restorativeness, although with different weights for parks and squares. We found that perceived quality of a space is a key predictor of its restorativeness. This means that maintaining parks and town squares is a relevant task given that they contribute to reducing cognitive overload, increasing sustainability, and facilitating health care in urban settings.

## Introduction

Today, more than half of the world’s population lives in cities, and forecasts point to an increase of three billion by 2050 (UNPF, 2018). However, this growth could lead to socio-environmental problems such as pollution, waste production, increasing inequality, and declining quality of life. It is therefore essential to increase the efficient use of resources, sustainable land distribution, and the protection of natural ecosystems in cities. In this context, urbanization has the potential to mark a new balance between economic growth, social welfare, and environmental care; the pillars of sustainability (UNPF, 2014). Among the objectives of Agenda 2030 on Sustainable Development, the 11th is focused on actions in increasingly urbanized societies to guarantee a healthy life (physical, social, and mental) that promotes well-being and favors the development of more liveable cities. Promoting equal access to the benefits arising from the use of public resources is also among these objectives. These benefits include the cognitive restorativeness that occurs when we interact with natural elements.

In order to alleviate increasing inequality and enhance environmental quality in cities, it is necessary to maintain and improve public spaces. It is therefore vital to understand how users of parks or public squares value these physical environments to contribute to the improvement of urban policies, planning and design, as well as the architecture of urban green spaces. Romice et al. (2017) pointed out that the professionalization in design has generated a belief among the population that problems related to urban design should be resolved by policy, legislation, and management. Against this background, Dempsey and Burton (2012) indicated that community movements, supported by research programs on space maintenance, can connect citizen participation with the design and management of open spaces. Therefore, more evidence is needed on how environmental characteristics perceived by users can affect the restorative capacity of open urban spaces.

### Restorativeness in Public Spaces

The benefits of contact with nature have been repeatedly established in both natural and human-made settings (McMahan and Estes, 2015; Honold et al., 2016). There is a need to return to the natural and, failing that, to increase opportunities to access the benefits of nature in urban contexts (Staats et al., 2010; Emfield and Neider, 2014). Moreover, in perceptions of public spaces, there is great consensus that restorativeness is associated with certain psychological benefits (Hartig et al., 2003; Herzog et al., 2003; Galindo and Hidalgo, 2005).

Restorativeness is defined as a process by which diminished psychological resources, often caused by stress or emotional fatigue, are restored (Hartig et al., 2001). The restorative effect is evidenced by attention and specifically by directed attention, since it involves a greater demand of resources compared with involuntary attention, the latter being able to induce experiences of relaxation while not requiring effort. In order to reduce the wear and tear of directed attention, environments or tasks that imply a lower demand are required. The theory on restorative experience developed by Kaplan and Kaplan (1989) is based on the influence of natural environments and the cognitive benefits that these spaces can favor (Ulrich, 1983; Kaplan and Kaplan, 1989; Grinde and Patil, 2009). Korpela and Hartig (1996) developed a model in which a restorative environment must have the characteristics of (a) avoidance, allowing users of public spaces a cognitive and psychological distance from everyday life; (b) compatibility, so that users will be able to carry out actions or make decisions based on two aspects: individual objectives or inclinations and actions required by the environment where the space is located; (c) fascination, which causes interest in users but without activating voluntary attention, so requiring a low attentional effort; (d) extension, which unifies sensations of connection and reach that spaces can have on users, making it easier for users to feel immersed in the environment and be predictable; and (e) coherence, referring to the impact that the physical arrangement of the elements causes on users.

Numerous studies have compared the restorative effect of natural spaces with urban spaces and have observed that urban spaces have a lower impact on cognitive and physiological restorativeness (Hartig and Evans, 1991; Franěk et al., 2018; Grassini et al., 2019; Ojala et al., 2019). Yet, to the extent that they have natural elements, urban spaces also contribute to cognitive restorativeness (Hernández and Hidalgo, 2005; San Juan et al., 2017). Urban environments can lead to an overload of stimulation and sustained attentional activation, which is why urban spaces with restorative capacity can be key to citizens’ health, especially, considering that urban spaces with high capacities for restorativeness can be accessible every day and easy to visit for people who live in cities. Indeed, there is ample evidence of the potential of urban spaces for restorativeness, such as museums (Kaplan et al., 1993), houses (Scopelliti and Giuliani, 2004), gardens (Twedt et al., 2016), botanical gardens (Carrus et al., 2017), historical-artistic sites (Scopelliti et al., 2019), or urban landscapes (Karmanov and Hamel, 2008). In this sense, Staat et al. (2016) highlighted the importance of examining different types of spaces to make comparisons between urban and natural ones (for example, green paths vs. busy streets).

In summary, the impact of the presence of natural elements on the restorative capacity of spaces has been consistently demonstrated in various investigations. This restorative capacity has been shown in natural settings and in urban ones where natural elements are present, especially trees and plants. However, while in natural spaces, there is little or no presence of elements built by humans; urban spaces in addition to natural elements contain varying degrees of built elements. Therefore, two questions immediately arise: Is the restorative capacity of urban spaces an exclusive product of the presence or absence of natural elements? Or is restoration also related to the characteristics of built elements?

Analyzing the joint contributions of natural and built elements, which are characteristics of parks and squares, is of interest for promoting the restorative capacity of public spaces. Research in this area has combined the role of urban nature with other variables such as type of leisure activities, the environment in which spaces are located, and the social context (Staats et al., 2010). A multiple influence has been observed. However, there is not enough evidence on whether the specific characteristics of these spaces, the way in which people perceive or do not perceive these characteristics, have an impact on their restorative effect. Therefore, it is necessary to analyze to what extent the characteristics or attributes of built elements contribute to the restorative power of squares and parks.

### Perceived Environmental Quality

The analysis of spaces’ characteristics and attributes and especially how they contribute to improving users’ lives has been carried out fundamentally from the concept of perceived environmental quality. This is a concept that addresses the physical conditions necessary to make a space habitable and improve people’s quality of life. However, it also involves linking psychological processes, as it can be approached from the evaluation that people make of a place. In short, environmental quality makes a tangible link between environments and people (Bonnes et al., 1997; Bonaiuto et al., 1999, 2003, 2006; Andrade et al., 2012; von-Breymann and Montenegro-Montenegro, 2019).

The impact of physical conditions, i.e., the quality of spaces, on various aspects of the interaction between human beings and their environment has been noted in evaluations of places related to residential satisfaction, place attachment, or habitability (Amérigo and Aragonés, 1990, 1997). Attributes such as noise level, neighborhood attractiveness, accessibility, and aspects related to maintenance explain satisfaction and attachment in neighborhoods (Amérigo and Aragonés, 1990, 1997; Ruiz and Hernández, 2014; Ruiz et al., 2019). In this direction, there is a frequent association between elements of environmental quality and well-being with several studies on the effects of urban design (Kleinert and Horton, 2016; Mangone, 2018), landscapes (Souter-Brown, 2015) or contact with nature (Corral-Verdugo et al., 2011; da Luz Reis et al., 2011). Other issues associated with environmental quality have been the perception of safety and security linked to the physical aspects of environments (Sautkina, 2007) or the emotional qualities associated with sound environments (Guillén and López-Barrio, 2007).

In summary, research into environmental quality and its effects on users’ preferences and uses of public spaces have indicated that increases in quality are linked to increases in preference and satisfaction. However, little is known about the effect that the quality of built elements has on the restorative capacity of spaces, especially as it is in built urban spaces that people conduct their daily lives and where they really connect with the city in which they live. As indicated by Van den Berg et al. (2007), we should investigate design and urban planning solutions in cities and their green spaces that support restorativeness. Along these lines, Peschardt and Stigsdotter (2013) indicated a lack of knowledge about which specific characteristics of urban green spaces are associated with restorativeness. Therefore, this paper aims to provide new information on the effect that perceived environmental quality has on users’ restorative experiences in these spaces.

One approach to the study of variables related to perceived environmental quality was carried out by Bonaiuto et al. (2019) based on the concept of urban reputation. This research shows that the evaluation of urban environments is multidimensional. The authors propose an assessment procedure made up of 27 subscales that are grouped into 12 dimensions. The results obtained show the importance of elements related to environmental quality in terms of design, care or maintenance, and social interaction.

Other research shows the importance of sensorial elements linked to hearing and smell. According to Emfield and Neider (2014), the images and sounds of nature are more relaxing than urban sounds. However, studies such as those by Krzywicka and Byrka (2017) and Payne et al. (2020) found a restorative potential of soundscapes in urban parks, despite the juxtaposition of nature sounds (such as birds) and urban sounds (such as traffic). Regarding the olfactory sense, some works go deeper into the role it can play in urban identity (Henshaw et al., 2016). In particular, Quercia et al. (2018) suggested the connection of odors with basic emotions and cognitive associations, specifically showing that unpleasant odors have a greater impact on autonomous activity, as well as participants’ preference for hedonistic odors. In general, it is observed that research that has taken into consideration “non-visual” sensorial elements is inconclusive, as well as being scarce when linked to restorativeness.

### Promising Restorative Urban Areas: Parks and Town Squares

Interest in studying green spaces in cities is increasing (Peschardt and Stigsdotter, 2013; White et al., 2013; Wood et al., 2018). Parks in urban areas influence self-informed psychological restorativeness, and as Wood et al. (2018) pointed out, the facilities in these spaces and their biodiversity are influential features of self-informed psychological restorativeness. Tyrväinen et al. (2014) and Vander Berg et al. (2014) suggest that the size of green spaces and their physical characteristics are relevant aspects for restorativeness. In general, the use of parks is associated with benefits such as tranquility, solitude, beauty, health, recreation, public life, and identity with the community (Kocs, 2013). Similarly, views from windows, which include views of natural environments also have restorative effects (Masoudinejad and Hartig, 2020). The experimental study presented by Honold et al. (2016) also showed the positive effect that urban nature has on health. Likewise, Hernández and Hidalgo (2005) verified the restorative effect of the presence of nature in photographed urban scenes. For all these reasons, it can be concluded that introducing natural elements in public spaces such as parks or squares is a way of enriching urban environments and increasing their restorative potential.

While parks play an important role in improving restorativeness and health in general, squares can be equally important. San Juan et al. (2017) carried out a study focused on urban squares where the presence of natural elements (grass, trees, water), architecture (variation of the surrounding buildings), coherence, and mystery were considered. The results of the work supported the restorative effect of squares, improving people’s psychological state after passing through an urban square. However, results showed no restorative differences according to the greenery. The authors argued that it is possible that, in urban environments, elements such as greenery or water may have less influence.

By contrast, in another study on urban squares, Lorenzo et al. (2016) confirm the relationship between users’ preferences and amount of vegetation. However, in this study, the role that the physical structures or the activities of the users of squares can play was not considered. Another aspect corroborated by previous studies (Purcell et al., 2001; Galindo and Hidalgo, 2005; Tenngart Ivarsson and Hagerhall, 2008) is that expected restorativeness from these spaces will influence users’ preferences.

Based on a literature review, the general aim of this study is to check whether elements of environmental quality perceived by users of public open spaces favor restorativeness. Specifically, we first hypothesize that the perceived quality of physical elements of parks and squares is positively related to perceived restorativeness. A second hypothesis is that parks generate higher levels of perceived restorativeness than squares.

## Materials and Methods

### Participants

This research involved 519 users of 32 urban public spaces—squares and parks—with free use. All participants were residents on the Island of Tenerife (Spain). This sample had an age range from 18 to 87 years old, with an average of 42.6 years old (*SD* = 15.75). Table 1 presents the sociodemographic data of the study participants.

**Table 1:** Percentage of sociodemographic variables.

Gender	Men	44.3%
	Women	55.7%
Educational level	No studies	2.7%
	Primary education	16.7%
	Secondary education or vocational training	36.3%
	Studying at university	7.2%
	Completed university studies	37.1%
Current work	Working	64.2%
	Unemployed	13.1%
	Studying in some way	11.6%
	Retired	11.1%
Marital status	Married or living with their partner	64.2%
	Single and/or not living with a partner	24.1%
	Separated or divorced	8.1%
	Widowed	3.6%

### Spaces Included in the Study

**Figure 1:**
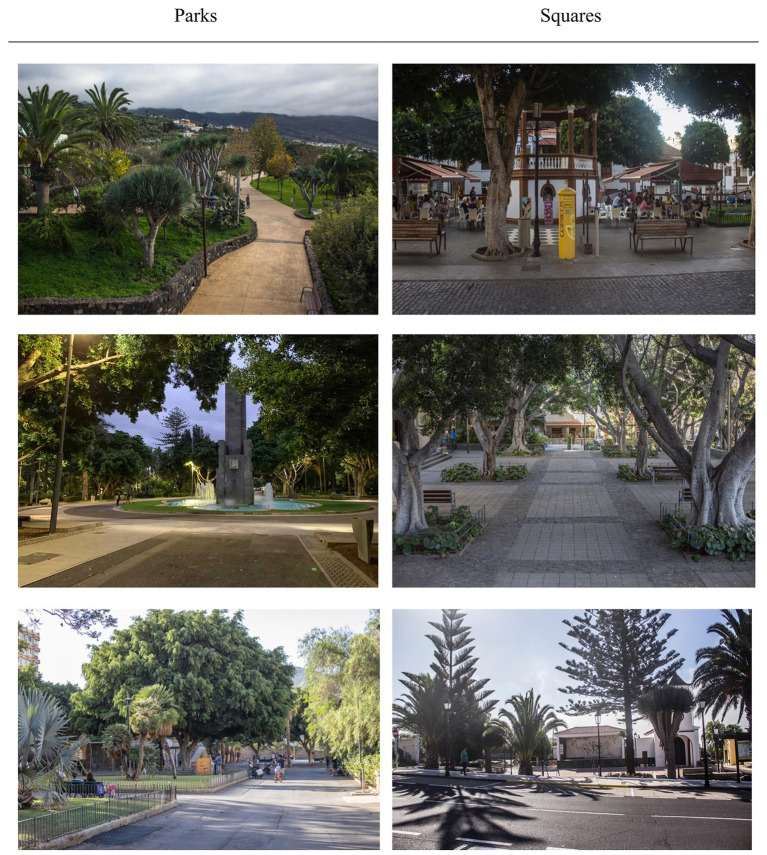
Photographs of parks and squares.

The public spaces evaluated are located in urban areas with populations of at least 3,000 inhabitants on the island of Tenerife (Spain). Figure 1 shows, through photographs, the various parks and squares under study (three squares and three parks).

The 32 spaces that were the subject of this research were evaluated by an expert observer using a validated tool (Rosales et al., 2019). This tool makes it possible to check the quantity and quality of the physical elements present in a public space according to 23 indicators, which are grouped into three main dimensions: architectural, functional, and contextual. In addition to an evaluation of each dimension, it is possible to obtain an overall evaluation combining all three dimensions.

The overall result of the evaluation of urban sites included in this work showed no differences [*t*(30) = −1.04, *p* > 0.05] between the two types of spaces—20 squares and 12 parks. Also, it was tested whether there were significant differences between squares and parks for each of the dimensions. No significant differences were found for the functional and contextual dimensions with both types of spaces showing a similar evaluation in functional and contextual elements. Thus, at the functional level, the squares and parks in this study were characterized by a shortage of toilets, the presence of traditional waste collection bins, the presence of some sculptures in the interior, having mostly shared benches and outdoor seating, and including prominent signage on regulations and flora. On a contextual level, both spaces had a good level of lighting at night, an adequate level of security due to the presence of surveillance signs, visibility from the outside, access for security vehicles and the absence of mostly dark areas, and a good level of cleanliness and physical order. However, in the architectural dimension, there were differences between squares and parks [*t*(30) = −5.11, *p* > 0.001]. In Table 2, a detailed description of the architectural elements for each type of urban space is presented. We tested whether each architectural feature was different depending on the type of public space and found that green and/or landscaped areas were larger in parks than in squares [*t*(30) = −4. 63, *p* < 0.001], the type of vegetation was also larger [*t*(30) = −2.63, *p* < 0.05], and the size distribution of areas for children, for sports, and for animals was significantly larger in parks than in squares [*t*(30) = −7.28, *p* < 0.001] as well (Table 2).

**Table 2:** Description of evaluated architectural elements of squares and parks through the observation tool.

Architectural features	Architectural elements	Square		Parks	
		Description	M^*^	Description	M^*^
Green and/or landscaped areas	Size	Small (<100 m^2^)	3.83	Large (>500 m^2^)	7.22
Type of vegetation	Lawn—grass Flowers—plants Shrubs Trees	Small variety of different vegetation types	7.0	Wide variety of different vegetation types	9.38
Children’s, sports and animal areas	Size	Small (<100 m^2^)	1.78	Medium (100—500 m^2^) and Large (>500 m^2^)	6.8
Accesibility	Open/closed enclosure Presence of private/transitable areas Presence of steps Access and transit for persons with reduced mobility	Open spaces, 100% walkable, with stairs and few limitations for people with reduced mobility	5.98	Open spaces, with impassable areas, no steps, and some limitations for people with reduced mobility	6.78
Perspective—sense of spaciousness	Enclosed by: Natural elements Buildings Mixed	Mixed enclosure	5	Enclosed by natural elements	4.2
Pedestrian circuit in the public space	Pavement condition Visually attractive for walking Difficulty of walking Weather protection elements (e.g., high-canopy trees, pergolas, etc.)	Adequate pavement with no major walking difficulties. Visually unattractive with some protective elements	6.25	Pavement could be improved with some walking difficulties. Very visually attractive with numerous protective elements	5.7

* Average rating of architectural features (scale of 1–10).

The first column presents the general architectural features included in the validated tool (Rosales et al., 2019), and the second column presents the architectural elements assessed for each of the architectural features. The next two columns—squares and parks—show, on the one hand, the description for both types of space for each architectural feature evaluated and the average score obtained for each architectural feature.

Based on these findings, analyses were carried out according to the type of space. Thus, in the results of this research, a subsection is presented for squares and another for parks.

### Study Design

The design of this study is predictive (Ato et al., 2013) as its purpose is to analyze the existence or not of a relationship between variables in order to determine or explain behavior. For this purpose, a survey methodology was used by which participants evaluated the spaces. We used four variables focused on perceived environmental quality: design of spaces, care of spaces, social interaction, and presence of sensorial elements (predictive variables). Additionally, the perceived restorativeness was evaluated (criterion variable).

### Materials and Instruments

A questionnaire was developed in three parts: (1) scale of perceived environmental quality, (2) scale of perceived restorativenes, and (3) sociodemographic variables.

The scale of perceived environmental quality is an adaptation based on the scales proposed by Ruiz and Hernández (2014), Bonaiuto et al. (2019), and Ruiz et al. (2019). It is composed of four dimensions with 21 items in total, with a Likert-type response scale from one to five, with 1 “Totally disagree” and 5 “Totally agree.” Specifically, this scale included the following variables:

-*Design of spaces subscale* is composed of seven items and whose internal consistency, measured with Cronbach’s α, was 0.72. This evaluation includes aspects related to signposting, accessibility, and waste disposal, as well as the presence of green spaces, places for free use, and aspects of the layout. For example, on this scale, the following items are used: “The different areas of this space are well signposted and connected” or “The transit or passage areas and interior paths of this place are wide enough.”
-*Care of space subscale* consists of six items and with an internal consistency of 0.84. Care of space is understood as the general conditions of maintenance and cleanliness of the place, both green areas and architectural elements, and is related to the external appearance of public spaces. Among the items included in this scale are, for example: “The maintenance of green and nature areas is correct” or “The level of cleanliness of this place, in general, is adequate.”
-*Social interaction subscale* is composed of four items with an internal consistency of 0.77. Social interaction is defined as the perceived quality of relationships with other users in public spaces and the possibilities of contact that these spaces promote. An example of the issues raised on this scale is “The attitude of users of this place makes me feel comfortable.”
-*Presence of sensorial element subscale* has four items and with an internal consistency of 0.75 This instrument includes aspects related to auditory and olfactory senses. Some examples of the items used are: “In this place, you can appreciate pleasant sounds” or “In this place, you can perceive pleasant smells.”

The Restorative Capacity scale, translated into Spanish and used by Ruiz and Hernández (2014) and Negrín et al. (2017) from the original scale proposed by Berto (2005), which, in turn, was developed as a reduced version of Korpela and Hartig’s (1996) Perceived Restorativeness Scale (PRS), is composed of five items whose response scale is Likert type from one to five, with 1 being “Totally disagree” and 5 “Totally agree.” The internal consistency was 0.82. For example, this scale includes the following items: “This place lets me forget my everyday responsibilities, feel relaxed, and lose myself in my own thoughts” or “I feel comfortable here because it’s easy to find my way around this place.”

With respect to sociodemographic variables, information was collected from participants on their gender, educational level, current work, and marital status.

### Procedure

Participants answered the questionnaire inside or near the public space that was to be evaluated. A group of five experienced interviewers were responsible for collecting the data. For this, the interviewers collected the data, in public spaces (squares and parks) at different times of the week (weekday vs. weekend) and at different time slots (morning/evening). Data were collected from January to early March 2020. For this work, interviewers used both the paper and pencil and the digital version of the questionnaire, using their own phone or tablet for the latter.

Before completing the questionnaire, the interviewers informed participants of the objective of the research and explained to them why their collaboration was essential. They also guaranteed the anonymity of participants’ responses and confidentiality of the information provided. To complete the questionnaire, the interviewer first read out the various items to the participant. Second, based on their responses, the interviewer marked the answers in the printed or digital version of the questionnaire. Finally, participants were thanked for their collaboration, and each participant was asked to indicate if he or she consented to the use of his or her answers for research purposes. The completion time ranged from approximately 20–40 min.

### Data Analysis

Data analysis was carried out using IBM SPSS Statistics software, version 21. In this study, the internal consistency of the different scales was calculated. Moreover, the average for each variable was calculated from the different items that make up the various subscales used. In addition, the descriptive statistics (mean, standard deviation, and response range) of all the variables and the correlations between them were calculated. Several one-factor analyses of variance (ANOVA) were conducted to examine whether there were differences among participants based on sociodemographic data. Finally, it was checked, through a regression analysis, if the elements of perceived environmental quality favored perceived restorativeness.

## Results

We analyze normality based on typical scores and multivariate outliers with the Mahalanobis distance, eliminating five outliers. The remaining analyses were carried out with 514 valid cases, of which 331 participants were interviewed inside or near squares and 183 participants inside or near parks.

First, we compared the scores of parks and squares on the predictor variables and on the criterion variable. Table 3 shows the differences between the two types of public spaces in three of the predictor variables (design of spaces, care of spaces, and presence of sensory elements) and in the criterion variable (perceived restorativeness) of the research.

**Table 3:** ANOVAs PV and CV.

		*M*	*df*	*F*
Design of spaces	Squares	3.56	(1,513)	90.39^***^
	Parks	4.14		
Care of spaces	Squares	3.26	(1,513)	19.09^***^
	Parks	3.64		
Social interaction	Squares	3.63	(1,513)	3.71
	Parks	3.78		
Presence of sensorial elements	Squares	3.23	(1,513)	56.67^***^
	Parks	3.85		
Perceived restorativeness	Squares	3.47	(1,513)	45.08^***^
	Parks	4.02		

*** *p* < 0.001.

### Sociodemographic Variables and Predictors: Squares

We analyzed the descriptive statistics for the different study variables focused on perceived environmental quality: design of spaces, care of spaces, social interaction, and presence of sensorial elements. The correlation between the variables in this study are also calculated. In Table 4, we present the descriptive statistics and the correlations between the predictor variables.

**Table 4:** Descriptive statistics and correlations of predictor variables for squares.

	*M*	Standard deviation (*SD*)	1	2	3	4
1. Design of spaces	3.56	0.68	-			
2. Care of spaces	3.26	0.89	0.45^**^	-		
3. Social interaction	3.63	0.80	0.44^**^	0.22^**^	-	
4. Presence of sensorial elements	3.24	0.86	0.40^**^	0.29^**^	0.47^**^	-

** *p* < 0.01.

Table 4 shows how the design of spaces is significantly and positively related to the care of spaces, social interaction, and presence of sensorial elements. The care of spaces is also significantly and positively related to social interaction and presence of sensorial elements. Social interaction is significantly and positively related to the presence of sensorial elements.

Likewise, we analyzed whether there were differences in the predictor variables (design of spaces, care of spaces, social interaction, and presence of sensorial elements) according to the sociodemographic data collected in the questionnaire (gender, educational level, current work, and marital status).

-*Gender*: No differences were identified for design of spaces, care of spaces, and social interaction. However, there were differences in the predictor variable presence of sensorial elements [*F*(1,329) = 9.26; *p* < 0.01]. Specifically, men have a higher valuation for sensorial elements (3.40) in one type of spaces—squares—compared with women (3.11).
-*Educational level*: No differences were identified for care of spaces and presence of sensorial elements. However, for the predictor variables design of spaces [*F*(4,326) = 2.43; *p* < 0.05] and social interaction [*F*(4,326) = 2.71; *p* < 0.05], significant differences were observed. In this sense, when analyzing these in greater depth by means of tests with Tukey’s HSD adjustment, it is observed that for the design of spaces, the differences disappear, while for social interaction, differences are observed between users with primary education (3.79) and those with university studies (3.44).
-*Current work*: No differences were identified for the design and care of spaces. Differences were found for social interaction [*F*(3,327) = 7.89; *p* < 0.001] and presence of sensorial elements [*F*(3,327) = 5.09; *p* < 0.01]. Specifically, from tests with Tukey’s HSD adjustment, differences are observed in the perception of social interaction in squares between retired participants (4.11) and the rest of the groups (working, 3.55; unemployed, 3.64; studying in some way, 3. 4, in terms of the presence of sensorial elements). Using Tukey’s HSD adjustment test, differences are observed, on the one hand, between those who are retired (3.54) compared with those who are working (3.19) and those who are studying (2.92) and, on the other hand, between those who are unemployed (3.45) and those who are studying (2.92).
-*Marital status*: no differences were identified for design of spaces, care of spaces, and presence of sensorial elements. However, there are differences in social interaction [*F*(3,327) = 4.09; *p* < 0.01]. In particular, from Tukey’s HSD adjustment test, it is observed that widowed participants (4.07) rate the social interaction of the spaces more highly than single users and/or those not living with a partner (3.40).

### Sociodemographic Variables and Predictors: Parks

We calculated the descriptive statistics for design of spaces, care of spaces, social interaction, and presence of sensorial elements. Furthermore, the correlations between the independent variables in this study were calculated. In Table 5, we present the descriptive statistics and the correlations among the predictor variables.

**Table 5:** Descriptive statistics and correlations of predictor variables for parks.

	*M*	Standard deviation (*SD*)	1	2	3	4
1. Design of spaces	4.14	0.64	-			
2. Care of spaces	3.64	1.01	0.65^**^	-		
3. Social interaction	3.77	0.78	0.40^**^	0.33^**^	-	
4. Presence of sensorial elements	3.86	0.94	0.53^**^	0.48^**^	0.37^**^	-

** *p* < 0.01.

Table 5 shows that, in parks, the study’s predictor variables are significantly and positively related to each other, just as they are in squares.

Additionally, the same as for the squares, we analyzed whether there were differences in the predictor variables (design of space, care of spaces, social interaction, and presence of sensorial elements) based on the sociodemographic data (gender, educational level, current work, and marital status).

-*Gender*: No differences were observed in the variable design of spaces, care of spaces, social interaction, and presence of sensorial elements.
-*Educational level*: No differences were identified for design of spaces, care of spaces and social interaction. By contrast, for the presence of sensorial elements [*F*(3,179) = 4.24; *p* < 0.01] significant differences were observed. Specifically, when analyzing these differences in greater depth by means of Tukey’s HSD test, it is observed that there are differences for the presence of sensorial elements between participants who are studying at university (3.08) compared with those who have secondary education or vocational training (4.03) and those who have completed university studies (3.89).
-*Current work*: As with the squares, no differences were identified for the design of spaces and care of spaces. However, there are differences in social interaction [*F*(3,179) = 3.19; *p* < 0.05] and the presence of sensorial elements [*F*(3,179) = 3.47; *p* < 0.05]. Specifically, based on Tukey’s HSD test, it is observed, on the one hand, that the differences in social interaction disappear. On the other hand, regarding the presence of sensory elements, the group of participants who are studying (3.25) has a lower perception of this type of elements compared with those users who are working (3.92) or unemployed (4.01).
-*Marital status*: No differences were identified for the variables of design of space, care of spaces, social interaction, and presence of sensorial elements.

### Perceived Restorativeness: Squares and Parks

We calculate the descriptive statistics for the perceived restorativeness. Moreover, we calculate the correlation between the variables (predictors and criterion) in this study. In Table 6, we present the descriptive statistics and the correlations between the dependent variable and the predictor variables.

**Table 6:** Descriptive statistics and correlations of the dependent variable for squares and parks.

		*M*	Standard deviation (*SD*)	1	2	3	4
Perceived restorativeness	Squares	3.47	0.90	0.55^**^	0.33^**^	0.57^**^	0.39^**^
	Parks	4.02	0.87	0.65^**^	0.65^**^	0.48^**^	0.52^**^

1. Design of spaces; 2. Care of spaces; 3. Social interaction; 4. Presence of sensorial elements.

** *p* < 0.001.

Table 6 shows that the perceived restorativeness for both squares and parks are significantly and positively related to the predictor variables (design of spaces, care of spaces, social interaction, presence of sensorial elements).

Furthermore, we examined whether there were differences in the criterion variable (perceived restorativeness) based on the sociodemographic data (gender, educational level, current work, and marital status).

-*Gender*: No gender differences were identified in either of the two types of spaces—squares and parks.
-*Educational level*: No differences were identified for perceived restorativeness between participants surveyed inside or near the squares. However, significant differences were found in parks [*F*(3,179) = 2.93; *p* < 0.05]. When analyzing these differences in greater depth by means of Tukey’s HSD test, it is observed that they disappear.
-*Current work*: No differences were identified for either squares or parks.
-*Marital status*: There are no differences in perceived restorativeness between the participants surveyed in either type of space, squares, or parks.

Likewise, based on the correlations observed between predictor variables (design of space, care of spaces, social interaction, presence of sensorial elements) and the perceived restorativeness, we performed a multiple linear regression with the stepwise method for both types of urban public spaces.

With respect to the squares, we assessed whether the sociodemographic variables (gender, educational level, current work, and marital status) as well as the predictor variables explain perceived restorativeness. This analysis yields three models where, in the third one, social interaction, design of spaces, and care of spaces predict the perception of restorativeness significantly [*F*(3,327) = 87.01; *p* < 0.001]. Thus, neither the presence of sensorial elements nor the sociodemographic variables predict the value of our criterion variable.

This final model complies with the assumptions of normality, non-colinearity and independence of the residual. Specifically, the assumption of normality has been checked from an analytical study of the normality of the residuals by means of the nonparametric Kolmogorov-Smirnov test (Z of K–S = 0.76; *p* > 0.05). Regarding the assumption of noncolinearity, both the tolerance statistic and the variance inflation factor (VIF) statistic were used as the procedure for detecting multicolinearity. The results of these are presented in Table 7, observing that for the three independent variables of the model (design of spaces, care of spaces, and social interaction), the tolerance statistic is greater than 0.10, and the variance inflation factor (VIF) is less than 10. Furthermore, in the definitive model, through the Durbin-Watson statistic test, the assumption of independence of the residual is fulfilled, as a value between 1.5 and 2.5 (1.78) is obtained.

**Table 7:** Output of the perceived environmental quality regression on perceived restorativeness—squares.

	Non-standardized coefficients		Standardized coefficients and *p*-value	95% confidence Interval		Partial correlations	Colinearity statistics	
	*B*	*SE*	*β*	Lower limit	Higher limit		Tolerance	VIF
Constant	−0.33	0.22						
Social interaction	0.46	0.05	0.41^***^	0.35	0.56	0.44	0.81	1.24
Design of spaces	0.43	0.06	0.33^***^	0.30	0.56	0.34	0.68	1.48
Care of spaces	0.09	0.05	0.09^*^	0.01	0.19	0.11	0.80	1.25
Adj R^2^	0.44							

* *p* < 0.05;

*** *p* < 0.001.

Once the assumptions of the regression analysis were verified, we observed that three of the four independent variables showed a statistically significant weight. Table 5 also includes the standardized coefficients (β), *p*-values and partial correlations.

In short, in the final model, social interaction, design of spaces, and care of spaces explain 44% of the variance in the restorative capacity perceived by participants interviewed inside or near the squares.

In relation to the parks, we analyzed whether sociodemographic variables (gender, educational level, current work, and marital status) and the predictor variables explain perceived restorativeness. This analysis yields six models where, in the last one, the level of studies studied at university, completed university studies, the design of spaces, the care of spaces, the social interaction, and the presence of sensory elements predict significantly the dependent variable [*F*(6,177) = 41.27; *p* < 0.001]. On the contrary, the rest of the sociodemographic variables do not predict the value of our criterion variable.

This final model, as with the squares, complies with the assumption of normality of the residuals (Z of K–S = 1.11; *p* > 0.05), the assumption of independence of the residual (Durbin-Watson statistic test = 1.97), and the assumption of noncolinearity (Table 8). Once the assumptions of the regression analysis were verified, we observed that two groups of users according to their level of education and all the independent variables showed a statistically significant weight. Table 8 also includes the standardized coefficients (β), *p*-values, and partial correlations.

**Table 8:** Output of the perceived environmental quality regression on perceived restorativeness—parks.

	Non-standardized coefficients		Standardized coefficients and *p*-value	95% confidence Interval		Partial correlations	Collinearity statistics	
	*B*	*SE*	*β*	Lower limit	Higher limit		Tolerance	VIF
Constant	0.13	0.31						
Completed university studies	−0.18	0.16	−0.06	−0.50	0.15	−0.08	0.91	1.10
Studying at university	−0.30	0.10	−0.16^**^	−0.49	−0.11	−0.23	0.91	1.10
Design of spaces	0.38	0.10	0.27^***^	0.19	0.56	0.29	0.49	2.02
Care of spaces	0.26	0.06	0.30^***^	0.15	0.37	0.32	0.53	1.89
Social interaction	0.26	0.06	0.23^***^	0.14	0.38	0.30	0.79	1.27
Presence of sensorial elements	0.14	0.06	0.15^*^	0.03	0.25	0.18	0.63	1.59
Adj R^2^	0.57							

* *p* < 0.05;

** *p* < 0.01;

*** *p* < 0.001.

In summary, completed university studies, studying at university, design of spaces, care of spaces, social interaction, and presence of sensorial elements explain 57% of the variance in the restorative capacity perceived by participants interviewed inside or near the parks.

## Discussion

This paper contributes to research into the effects that the perceived quality of public spaces can have on perceived restorativeness. The results obtained confirm our first hypothesis that the perceived quality of the physical elements of parks and squares is positively related to their restorative capacity perceived.

The results of the study show that the design and care of spaces, as well as the presence of sensorial elements are more present in parks than in squares. Valera et al. (2018) point out that the quality and good functioning of a public space can be assessed according to the diversity of uses it allows. In this sense, squares are wide, open public spaces, where we find elements such as benches, statues, monuments, etc. They usually serve as a cultural representation of cities, as well as a meeting place for people. In these spaces, users sit, stroll, or spend time, making them functional places that favor social contact. As for parks, they fulfill a recreational function, and this is reflected in their layout and elements. They generally have green areas (gardens and trees, water fountains, etc.) and offer a greater number of services and possibilities of use than squares (sports practice, playgrounds, etc.). This distinction means that the elements that make up parks are greater in number and more diverse. It is not surprising that the scores in the dimensions of design, care, and presence of sensorial elements are higher for parks. However, no differences are found in social interaction, concluding that both parks and squares offer equivalent possibilities to develop social life.

There are also differences according to the sociodemographic variables on the quality elements evaluated (design, care, social interaction, and presence of sensorial elements). However, when introducing the sociodemographic variables in a first step of the regression model on perceived restorativeness, the variance explained is low, if not null. The hypothesis of a greater perceived restorativeness capacity in parks than in squares is also confirmed. This result is attributed to the greater presence of green areas in parks. Tyrväinen et al. (2014) reported that even in short visits, urban forests (with a greater presence of nature) provide greater psychological benefits than large urban parks. Moreover, the perceived availability of nearby green spaces on their own is associated with improvements in self-reported quality of life (Hipp and Ogunseitan, 2011). Additionally, studies in immersive virtual environments have shown differences between parks and squares when manipulating physical aspects of the environment such as the enclosure of these areas. In the case of squares, an enclosed spatial layout positively affects restorativeness. However, in parks, this same condition is inversely related to restorative capacity (Tabrizian et al., 2018). In line with this work, it is worth noting that our results revealed that there are differences in the impact that environmental quality conditions have on perceived restorativeness. To test this effect, stepwise regression models were applied to control for the effect of sociodemographic variables. Specifically, we obtained two models in which different dimensions of environmental quality explain perceived restorativeness in urban open-use spaces. For squares, we observed that higher scores in social interaction and spaces design and care, predict higher perceived restorativeness level. The coefficients indicate a higher weight mainly of social interaction and design of spaces. Regarding parks, we observed that higher scores in spaces’ care and design, social interaction, and presence of sensorial elements lead to higher levels of perceived restorativeness. In this case, the greatest weight is given to the importance of park maintenance as the first factor. The comparison of the two models shows that there is a relationship between the elements of environmental quality and perceived restorativeness. The question that arises is to analyze whether these models are comparable. Both squares and parks have in common a significant weight in the design of spaces. These data are relevant if we consider other research on the role that the design of squares and parks plays on users’ preferences (Korpela and Hartig, 1996; Korpela et al., 2008, 2010) as well as on variables such as vitality (Anderson et al., 2017). This finding is in line with other studies that highlight that aspects related to design, such as the layout of urban streets, are associated with social interaction and a sense of community (Wood et al., 2008).

Regarding differences, it was observed that care of spaces is more important in parks than in squares. Given that parks have a greater presence of natural elements, whether lawns, gardens, or trees, it is assumed that the maintenance of these areas should be continuous over time. To the contrary, a lack of conservation is evident in a shorter period of time than in the case of built elements. In addition, recreational areas, such as a playground or a sports field, also require supervision to be kept in optimal conditions. These aspects of care are closely related to the perception of hazards and risks. In this sense, Valera et al. (2018) point out the effect that spaces’ care factors (vandalism, inadequate lighting, or the presence of litter, for example) have on perceptions of insecurity and a subsequent reduced use of parks and squares. In this same study, the authors allude to the fact that the self-perceived most vulnerable groups (the elderly and women) may be less present in order to avoid risks or dangers. On the other hand, it is concluded that maintaining parks in optimal conditions invites the use of these spaces, favoring a more democratic use, as well as the enjoyment of more restorative experiences.

Another difference observed between the regression models was that the presence of sensorial elements only contributes to explained variance in parks. We associate this result with the fact that sounds associated with nature tend to be perceived more favorably than urban sounds (Krzywicka and Byrka, 2017), as well as the association that certain “natural” smells (bees wax, summer air) have with basic hedonic emotions (Glass et al., 2014). In this case, we consider that the presence of green areas intensifies the sensorial experience, and therefore, it could explain why it has a greater weight in the predictive model of parks. Since the presence of sensorial elements has been less studied, it would be advisable to analyze in greater depth what contributions sensorial elements make to restorativeness, including perceptive and objective measures with respect to dimensions such as noise or air quality. Enhancing the positive effects of public spaces includes favoring opportunities like the enjoyment of a good climate in the region, the aesthetic experience, and generation of sensorial enjoyment (Gehl, 2014).

Finally, with regard to the differences between the predictive models for parks and squares, it was observed that in the case of squares, the dimension of social interaction is emphasized. On the one hand, this indicates that the function of exchange and social encounter contributes significant value to the perceived restorativeness. On the other hand, squares can be representative of a higher quality of urbanism, in that they reflect a greater experience of social life. All this makes the role of social interaction more relevant to urban life in squares, which is consistent with other works (Galindo and Hidalgo, 2005; Peschardt et al., 2012). However, despite the social dimension being relevant in the perceived restorativeness experience, it has been an issue scarcely addressed in previous studies (Collado et al., 2017). In addition to this effect on perceived restorativeness, the literature emphasizes that superficial contacts between neighbors and perceived cohesion within neighborhoods are influenced by the availability of green and public spaces (Kaczynski and Henderson, 2007; Hayley et al., 2014).

In conclusion, it is necessary to provide some indications regarding interventions to improve parks and squares. In this sense, a commitment to urban design is useful for both spaces. This type of intervention can produce, among other effects, facilitation of welfare activities, promotion of community life, increased perceptions of vitality, connection with others, and greater use of public spaces (Anderson et al., 2017). Regarding the promotion of physical activity, the impact of urban design has also been verified. Branas et al. (2011) and Cohen et al. (2014) pointed out that the inclusion of green areas or small parks in empty spaces is a facilitator of physical exercise. Another element of design that is suggested by these results is the inclusion of green areas. In accordance with other works, the presence of plants, water or trees provide restorative benefits (Hunter and Askarinejad, 2015). This measure would help urban squares to gain a greater restorative potential. Focusing, therefore, on the improvement of squares, it is also concluded that the inclusion of natural elements could favor sensorial elements that broaden the perceived restorativeness. Specifically, more effort should be invested in considering the sociopetal properties that tend to keep people together and encourage social interaction. This distinctive feature of squares favors urban vitality, a quality that places people at the center of the design of public spaces and can be associated with benefits such as an increased sense of belonging, neighborhood identity, or a sense of community.

These findings on spatial design and interaction go in the direction of the new urban planning strategies catalogued as New Urbanism (Calthorpe and Fulton, 2001). Public and pedestrian traffic, the size of neighborhoods, the proportion and distribution of housing and commercial areas, and the inclusion of green spaces and the preservation of trees are strategies that influence the development and maintenance of a sense of neighborhood (Youngentob and Hostetler, 2005). As well as fostering a sense of community, these strategies adopt a conservation role for the environment in building practices. As Gehl (2014) argues, public spaces must be vital places, and the presence of other people is a promise of social interaction. A dynamic city offers urban life where recreational and social activities take place, but also pedestrian traffic, so that it feeds back by attracting flows of people.

This study is not without its limitations. First, although data collection was carried out at similar times of the day to avoid fluctuations, collecting data at different times of the week (weekday vs. weekend), different time slots (morning/evening/night), and the time spent by interviewers at the sites (1–3 h) would have offered a more representative range of responses. Second, this is a transversal work that provides descriptive knowledge of the relationship between environmental quality and perceived restorativeness. However, longitudinal analysis would complement the results, allowing us to assess whether the frequency of attendance or use of public spaces has any effect on restorativeness.

Regarding future research lines, social interaction is associated with perceived restorativeness. Therefore, the role of place attachment, which implies the development of affective ties with public spaces, should be taken into account. It would also be valuable to note the differences according to the cultural framework in which studies take place. Likewise, it would be interesting to check the effect of the size of the public space on the restorative experience. In this sense, the works of Hunter and Askarinejad (2015) and Mayne et al. (2015) stand out, as they point out greater effects on physical and mental well-being in smaller parks than in larger ones. Furthermore, within the challenges of sustainability, in cities where atmospheric pollution, waste generation, and the greater demand for natural resources are more present, the intervention and integration of restorative spaces makes more sense. Therefore, it would be relevant to see to what extent small interventions in parks and squares have on the restorativeness of these spaces.

Our findings argue that cities offer opportunities for restorativeness through their public spaces. Specifically, it is verified that parks and squares with suitable designs and offering possibilities of social interaction can contribute experiences of perceived restorativeness to users. These perceived restorative experiences will promote the recovery of psychological resources such as attention, positive moods, and stress reduction, enabling a healthier life in cities.

## Data Availability Statement

The raw data supporting the conclusions of this article will be made available by the authors, without undue reservation.

## Ethics Statement

Ethical review and approval was not required for the study on human participants in accordance with the local legislation and institutional requirements. The patients/participants provided their written informed consent to participate in this study.

## Author Contributions

MR-R participated in the design of the instruments, supervision of data collection, data analysis, writing and revision of the text, and author of correspondence. CR participated in the design of the instruments, data collection and supervision, data analysis, and text writing and review. ML participated in the collection of data and the revision of the text. GM participated in the elaboration of the research project, supervision of data collection, and the writing of the text and its revision. BH participated in the whole process of elaboration and writing of the paper. All authors contributed to the article and approved the submitted version.

### Conflict of Interest

The authors declare that the research was conducted in the absence of any commercial or financial relationships that could be construed as a potential conflict of interest.

## References

[ref1] Amérigo M. Aragonés J. I. (1990). Residential satisfaction in council housing. J. Environ. Psychol. 10, 313–325. 10.1016/S0272-4944(05)80031-3

[ref2] Amérigo M. Aragonés J. I. (1997). A theoretical and methodological approach to the study of residential satisfaction. J. Environ. Psychol. 17, 47–57. 10.1006/jevp.1996.0038

[ref3] Anderson J. Ruggeri K. Steemers K. Huppert F. (2017). Lively social space, well-being activity, and urban design: findings from a low-cost community-led public space intervention. Environ. Behav. 49, 685–716. 10.1177/0013916516659108

[ref4] Andrade C. Lima M. L. Fornara F. Bonaiuto M. (2012). Users' views of hospital environmental quality: validation of the perceived hospital environment quality indicators (PHEQIS). J. Environ. Psychol. 32, 97–111. 10.1016/j.jenvp.2011.12.001

[ref5] Ato M. Lopez J. J. Benavente A. (2013). A classification system for research designs in psychology. Anales de Psicología 29, 1038–1059. 10.6018/analesps.29.3.178511

[ref6] Berto R. (2005). Exposure to restorative environments helps restore attentional capacity. J. Environ. Psychol. 25, 249–259. 10.1016/j.jenvp.2005.07.001

[ref7] Bonaiuto M. Aiello A. Perugini M. Bonnes M. Ercolani A. P. (1999). Multidimensional perception of residential environment quality and neighborhood attachment in the urban environment. J. Environ. Psychol. 19, 331–352. 10.1006/jevp.1999.0138

[ref83] Bonaiuto M. Ariccio S. De Dominicis S. Fornara F. Molinario E. Troffa R.. (2019). City reputation indicators (CRIs): measuring inhabitants’ city representation/indicadores de reputación urbana: midiendo la representación de una ciudad en sus habitantes. PsyEcology 10, 31–87. 10.1080/21711976.2018.1545348

[ref8] Bonaiuto M. Fornara F. Bonnes M. (2003). Indexes of perceived residential environment quality and neighbourhood attachment in urban environments: a confirmation study on the city of Rome. Landsc. Urban Plan. 65, 41–52. 10.1016/S0169-2046(02)00236-0

[ref9] Bonaiuto M. Fornara F. Bonnes M. (2006). Perceived residential environment quality in middle- and low-extension Italian cities. Revue Européenne de Psychologie Appliquée 56, 23–34. 10.1016/j.erap.2005.02.011

[ref10] Bonnes M. Bonaiuto M. Aiello A. Perugini M. Ercolani A. P. (1997). “A Transactional Perspective on Residential Satisfaction,” in Housing Surveys: Advances in Theory and Methods. eds. Despres C. Piché D. (Quebec, Canada: Crad Université Laval), 99–135.

[ref11] Branas C. C. Cheney R. A. MacDonald J. M. Tam V. W. Jackson T. D. Ten Have T. R. (2011). A difference-in-differences analysis of health, safety, and greening vacant urban space. Am. J. Epidemiol. 174, 1296–1306. 10.1093/aje/kwr273, PMID: 22079788 PMC3224254

[ref12] Calthorpe P. Fulton W. (2001). The Regional City: Planning for the End of Sprawl. Washington, DC: Island.

[ref13] Carrus G. Scopelliti M. Panno A. Lafortezza R. Colangelo G. Pirchio S.. (2017). A Different way to stay in touch with ‘Urban Nature’: the perceived restorative qualities of botanical gardens. Front. Psychol. 8:914. 10.3389/fpsyg.2017.00914, PMID: 28620335 PMC5450850

[ref14] Cohen D. A. Marsh T. Williamson S. Han B. Derose K. P. Golinelli D.. (2014). The potential for pocket parks to increase physical activity. Am. J. Health Promot. 28, S19–S26. 10.4278/ajhp.130430-QUAN-213 24380461 PMC4091959

[ref15] Collado S. Staats H. Corraliza J. A. Hartig T. (2017). “Restorative Environments and Health,” in International Handbooks of Quality-of-Life: Handbook of Environmental Psychology and Quality of Life Research. eds. Fleury-Bahi G. Pol E. Navarro O. (Switzerland: Springer International Publishing), 127–148.

[ref16] Corral-Verdugo V. Mirles-Acosta J. Tapia-Fonllem C. Fraijo-Sing B. (2011). Happiness as correlate of sustainable behavior: a study of pro-ecological, frugal, equitable and altruistic actions that promote subjective wellbeing. Hum. Ecol. Rev. 18, 95–104.

[ref17] da Luz Reis Tarcísio A. Barcelos A. (2011). “Green Spaces, Vegetation, and Well-Being in the Housing Environment,” in Urban Diversities — Environmental and Social Issues. eds. Bonaiuto M. Bonnes M. Nenci A. M. Carrus G. (Cambridge, MA: Hogrefe Publishing), 137–146, 255 Pages.

[ref18] Dempsey N. Burton M. (2012). Defining place-keeping: the long-term management of public spaces. Urban Forest. Urban Green. 11, 11–20. 10.1016/j.ufug.2011.09.005

[ref19] Emfield A. G. Neider M. B. (2014). Evaluating visual and auditory contributions to the cognitive restoration effect. Front. Psychol. 5:548. 10.3389/fpsyg.2014.00548, PMID: 24926279 PMC4046122

[ref20] Franěk M. Šefara D. Petružálek J. Cabal J. Myška K. (2018). Differences in eye movements while viewing images with various levels of restorativeness. J. Environ. Psychol. 57, 10–16. 10.1016/j.jenvp.2018.05.001

[ref22] Galindo M. P. Hidalgo M. C. (2005). Aesthetic preferences and the attribution of meaning: environmental categorization processes in the evaluation of urban scenes. Int. J. Psychol. 40, 19–27. 10.1080/00207590444000104

[ref23] Gehl J. (2014). Ciudades para la gente. Buenos Aires: Ediciones infinito.

[ref24] Glass S. T. Lingg E. Heuberger E. (2014). Do ambient urban odors evoke basic emotions? Front. Psychol. 5:340. 10.3389/fpsyg.2014.00340, PMID: 24860522 PMC4017720

[ref26] Grassini S. Revonsuo A. Castellotti S. Petrizzo I. Benedetti V. Koivisto M. (2019). Processing of natural scenery is associated with lower attentional and cognitive load compared with urban ones. J. Environ. Psychol. 62, 1–11. 10.1016/j.jenvp.2019.01.007

[ref27] Grinde B. Patil G. G. (2009). Biophilia: does visual contact with nature impact on health and well-being? Int. J. Environ. Res. Public Health 6, 2332–2343. 10.3390/ijerph6092332, PMID: 19826546 PMC2760412

[ref28] Guillén J. López-Barrio I. (2007). Criterios perceptivos en la valoración de la calidad sonora urbana. Revista de Psicología Soc. 22, 279–288. 10.1174/021347407782194416

[ref82] Hartig T. Evans G. W. (1991). Restorative effects of natural environment experiences. Environ. Behav. 23, 3–26. 10.1177/0013916591231001

[ref29] Hartig T. Evans G. W. Jamner L. D. Davis D. S. Gärling T. (2003). Tracking restoration in natural and urban field settings. J. Environ. Psychol. 23, 109–123. 10.1016/S0272-4944(02)00109-3

[ref30] Hartig T. Kaiser F. G. Bowler P. A. (2001). Psychological restoration in nature as a positive motivation for ecological behavior. Environ. Behav. 33, 590–607. 10.1177/00139160121973142

[ref31] Hayley C. H. Knuiman M. Divitini M. Foster S. Bull F. Giles-Corti B.. (2014). A Longitudinal analysis of the influence of the neighborhood built environment on walking for transportation: the RESIDE study. Am. J. Epidemiol. 180, 453–461. 10.1093/aje/kwu171 25117660

[ref32] Henshaw V. Medway D. Warnaby G. Perkins C. (2016). Marketing the ‘city of smells’. Mark. Theory 16, 153–170. 10.1177/1470593115619970

[ref33] Hernández B. Hidalgo M. C. (2005). Effect of urban vegetation on psychological restorativeness. Psychol. Rep. 96, 1025–1028. 10.2466/PR0.96.3.1025-1028 16173374

[ref34] Herzog T. R. Maguire C. P. Nebel M. B. (2003). Assessing the restorative components of environments. J. Environ. Psychol. 23, 159–170. 10.1016/S0272-4944(02)00113-5

[ref91] Hipp J. A. Ogunseitan O. A. (2011). Effect of environmental conditions on perceived psychological restorativeness of coastal parks. J. Environ. Psychol. 31, 421–429. 10.1016/j.jenvp.2011.08.008

[ref35] Honold J. Lakes T. Beyer R. van der Meer E. (2016). Restoration in urban spaces: nature views from home, greenways, and public parks. Environ. Behav. 48, 796–825. 10.1177/0013916514568556

[ref36] Hunter M. R. Askarinejad A. (2015). Designer's approach for scene selection in tests of preference and restoration along a continuum of natural to manmade environments. Front. Psychol. 6:1228. 10.3389/fpsyg.2015.01228, PMID: 26347691 PMC4541156

[ref37] Kaczynski A. T. Henderson K. A. (2007). Environmental correlates of physical activity: a review of evidence about parks and recreation. Leis. Sci. 29, 315–354. 10.1080/01490400701394865

[ref38] Kaplan S. Bardwell L. V. Slakter D. B. (1993). The museum as a restorative environment. Environ. Behav. 25, 725–742. 10.1177/0013916593256004

[ref39] Kaplan R. Kaplan S. (1989). The Experience of Nature: A Psychological Perspective. New York: Cambridge University Press.

[ref40] Karmanov D. Hamel R. (2008). Assessing the restorative potential of contemporary urban environment (s): beyond the nature versus urban dichotomy. Landsc. Urban Plan. 86, 115–125. 10.1016/j.landurbplan.2008.01.004

[ref41] Kleinert S. Horton R. (2016). Urban design: an important future force for health and wellbeing. Lancet 388, 2848–2850. 10.1016/S0140-6736(16)31578-1 27671666

[ref42] Kocs E. A. (2013). Finding Nature in the City: A Case Study of Ecological Restoration in an Urban Park. Ann Arbor: Dissertation Publishing.

[ref43] Korpela K. Hartig T. (1996). Restorative qualities of favourite places. J. Environ. Psychol. 16, 221–233. 10.1006/jevp.1996.0018

[ref44] Korpela K. Ylén M. Tyrväinen L. Silvennoinen H. (2008). Determinants of restorative experiences in everyday favourite places. Health Place 14, 636–652. 10.1016/j.healthplace.2007.10.008 18037332

[ref45] Korpela K. M. Ylén M. Tyrväinen L. Silvennoinen H. (2010). Favorite green, waterside and urban environments, restorative experiences and perceived health in Finland. Health Promot. Int. 25, 200–209. 10.1093/heapro/daq007, PMID: 20176589

[ref46] Krzywicka P. Byrka K. (2017). Restorative qualities of and preference for natural and urban soundscapes. Front. Psychol. 8:1705. 10.3389/fpsyg.2017.01705, PMID: 29046653 PMC5632731

[ref48] Lorenzo E. Corraliza J. A. Collado S. Sevillano V. (2016). Preference, restorativeness and perceived environmental quality of small urban spaces. Psyecology 7, 152–177. 10.1080/21711976.2016.1149985

[ref49] Mangone G. (2018). Exploring urban design strategies that maximize the benefits of urban nature for children's well-being. Ecopsychology 10, 216–227. 10.1089/eco.2018.0054

[ref50] Masoudinejad S. Hartig T. (2020). Window view to the sky as a restorative resource for residents in densely populated cities. Environ. Behav. 52, 401–436. 10.1177/0013916518807274

[ref51] Mayne S. L. Auchincloss A. H. Michael Y. L. (2015). Impact of policy and built environment changes on obesity-related outcomes: a systematic review of naturally occurring experiments. Obes. Rev. 16, 362–375. 10.1111/obr.12269, PMID: 25753170 PMC4789114

[ref52] McMahan E. A. Estes D. (2015). The effect of contact with natural environments on positive and negative affect: A meta-analysis. J. Posit. Psychol. 10, 507–519. 10.1080/17439760.2014.994224

[ref53] Negrín F. Hernández-Fernaud E. Hess S. Hernández B. (2017). Discrimination of urban spaces with different level of restorativeness based on the original and on a shorter version of hartig et al.’s perceived restorativeness scale. Front. Psychol. 8:1735. 10.3389/fpsyg.2017.01735, PMID: 29062293 PMC5640703

[ref54] Ojala A. Korpela K. Tyrväinen L. Tiittanen P. Lanki T. (2019). Restorative effects of urban green environments and the role of urban-nature orientedness and noise sensitivity: a field experiment. Health Place 55, 59–70. 10.1016/j.healthplace.2018.11.004 30502229

[ref85] Payne S. R. Nordh H. Hassan R. (2020). “Are Urban Park Soundscapes Restorative Or Annoying?” in 10th European Congress and Exposition on Noise Control Engineering 2015; May 3–June 3, 2015; Maastricht, Netherlands, 823–827.

[ref90] Peschardt K. K. Schipperijn J. Stigsdotter U. K. (2012). Use of small public urban green spaces (SPUGS). Urban For. Urban Gree. 11, 235–244. 10.1016/j.ufug.2012.04.002

[ref56] Peschardt K. Stigsdotter U. (2013). Associations between park characteristics and perceived restorativeness of small public urban green spaces. Landsc. Urban Plan. 112, 26–39. 10.1016/j.landurbplan.2012.12.013

[ref57] Purcell T. Peron E. Berto R. (2001). Why do preferences differ between scene types? Environ. Behav. 33, 93–106. 10.1177/00139160121972882

[ref58] Quercia D. Aiello L. M. Schifanella R. (2018). Diversity of indoor activities and economic development of neighborhoods. PLoS One 13:18. 10.1371/journal.pone.0198441 PMC6010263 29924816

[ref59] Romice O. Thwaites K. Porta S. Greaves M. Barboru G. Pasino P. (2017). “Urban Design and Quality of Life,” in Handbook of Environmental Psychology and Quality of Life Research. eds. Fleury-Bahi G. Pol E. Navarro O. (Switzeland: Springer), 241–273.

[ref60] Rosales C. Muinos G. Ríos M. L. Cabrera A. Febles M. N. Hernández B. y Lorenzo M. (2019). *Evaluación de las condiciones físicas de plazas, parques y jardines* [comunicación oral]. XV Congreso Internacional de Psicología Ambiental. Comunidad, recursos y sostenibilidad. San Cristóbal de La Laguna, Canarias, España.

[ref61] Ruiz C. Hernández B. (2014). Emotions and coping strategies during an episode of volcanic activity and their relations to place attachment. J. Environ. Psychol. 38, 279–287. 10.1016/j.jenvp.2014.03.008

[ref62] Ruiz C. Hernández-Fernaud E. Rolo-González G. Hernández B. (2019). Neighborhoods’ evaluation: Influence on well-being variables. Front. Psychol. 10:1736. 10.3389/fpsyg.2019.01736, PMID: 31417463 PMC6685347

[ref63] San Juan C. Subiza-Pérez M. Vozmediano L. (2017). Restoration and the city: the role of public urban squares. Front. Psychol. 8:2093. 10.3389/fpsyg.2017.02093, PMID: 29270139 PMC5725966

[ref64] Sautkina E. (2007). Environmental representations of safety from crime versus insecurity: a study in Lisbon. Revista de Psicología Social 22, 289–298. 10.1174/021347407782194362

[ref65] Scopelliti M. Carrus G. Boniauto M. (2019). Is it really nature that restores people? a comparison with historical sites with high restorative potential. Front. Psychol. 9:2742. 10.3389/fpsyg.2018.02742, PMID: 30745891 PMC6360171

[ref66] Scopelliti M. Giuliani M. V. (2004). Choosing restorative environments across the life span: a matter of place experience. J. Environ. Psychol. 24, 423–437. 10.1016/j.jenvp.2004.11.002

[ref67] Souter-Brown G. (2015). Landscape and Urban Design for Health and Well-Being: Using Healing, Sensory and Therapeutic Gardens. New York, NY: Routledge: Taylor & Francis Group.

[ref68] Staats H. van Gemerden E. Hartig T. (2010). Preference for restorative situations: interactive effects of attentional state, activity-in-environment, and social context. Leis. Sci. 32, 407–417. 10.1080/01490400.2010.510990

[ref89] Tabrizian P. Baran P. K. Smith W. R. Meentemeyer R. K. (2018). Exploring perceived restoration potential of urban green enclosure through immersive virtual environments. J. Environ. Psychol. 55, 99–109. 10.1016/j.jenvp.2018.01.001

[ref69] Tenngart Ivarsson C. Hagerhall C. M. (2008). The perceived restorativeness of gardens – assessing the restorativeness of a mixed built and natural scene type. Urban Forest. Urban Green. 7, 107–118. 10.1016/j.ufug.2008.01.001

[ref70] Twedt E. Rainey R. M. Proffitt D. R. (2016). Designed natural spaces: informal gardens are perceived to be more restorative than formal gardens. Front. Psychol. 7:88. 10.3389/fpsyg.2016.00088, PMID: 26903899 PMC4749713

[ref71] Tyrväinen L. Ojala A. Korpela K. Lanki T. Tsunetsugu Y. Kagawa T. (2014). The influence of urban green environments on stress relief measures: a field experiment. J. Environ. Psychol. 38, 1–9. 10.1016/j.jenvp.2013.12.005

[ref72] Ulrich R. (1983). “Aesthetic and Affective Response to Natural Environment,” in Behavior and the Natural Environment. eds. Altman I. Wohlwill J. F. (New York, NY: Plenum Press), 85–125.

[ref73] UNPF (2014). Ciudades y comunidades sostenibles. Available at: https://www.un.org/sustainabledevelopment/es/cities/ (Accessed September 25, 2020).

[ref74] UNPF (2018). Las Ciudades Seguirán Creciendo, Sobre Todo en los Países en Desarrollo. Available at: https://www.un.org/development/desa/es/news/population/2018-world-urbanization-prospects.html (Accessed September 20, 2020).

[ref75] Valera S. Pérez-Tejera F. Anguera M. T. Sicilia L. (2018). Evaluating the uses and environmental characteristics of 40 public parks and squares in Barcelona by means of systematic observation. PsyEcology 9, 118–151. 10.1080/21711976.2018.1432525

[ref76] Van den Berg A. E. Hartig T. Staats H. (2007). Preference for nature in urbanized societies: stress, restoration, and the pursuit of sustainability. J. Soc. Issues 63, 79–96. 10.1111/j.1540-4560.2007.00497.x

[ref77] Vander Berg A. E. Jorgersen A. Wilson E. (2014). Evaluating restoration in urban green spaces: does setting type make a difference? Landsc. Urban Plan. 127, 173–181. 10.1016/j.landurbplan.2014.04.012

[ref78] von-Breymann Montenegro-Montenegro (2019). Validation of a scale to measure perceived residential environment quality in a Latin American setting. Psyecology Bilingual. J. Environ. Psychol. 10, 217–256. 10.1080/21711976.2019.1579471

[ref79] White M. P. Pahl S. Ashbullby K. Herbert S. Depledge M. H. (2013). Feelings of restoration from recent nature visits. J. Environ. Psychol. 35, 40–51. 10.1016/j.jenvp.2013.04.002

[ref87] Wood E. Harsant A. Dallimer M. de Chavez A. C. McEachan R. R. C. Hassall C. (2018). Not all green space is created equal: biodiversity predicts psychological restorative benefits from urban green space. Front. Psychol. 9:2320. 10.3389/fpsyg.2018.02320 30538653 PMC6277587

[ref80] Wood L. Shannon T. Bulsara M. Pikora T. McCormack G. Giles-Corti B. (2008). The anatomy of the safe and social suburb: an exploratory study of the built environment, social capital and residents’ perceptions of safety. Health Place 14, 15–31. 10.1016/j.healthplace.2007.04.004 17576088

[ref81] Youngentob K. Hostetler M. (2005). Is a new urban development model building greener communities? Environ. Behav. 37, 731–759. 10.1177/0013916505275311

